# Anti-spike, Anti-nucleocapsid and Neutralizing Antibodies in SARS-CoV-2 Inpatients and Asymptomatic Individuals

**DOI:** 10.3389/fmicb.2020.584251

**Published:** 2020-10-19

**Authors:** Etienne Brochot, Baptiste Demey, Antoine Touzé, Sandrine Belouzard, Jean Dubuisson, Jean-Luc Schmit, Gilles Duverlie, Catherine Francois, Sandrine Castelain, Francois Helle

**Affiliations:** ^1^Department of Virology, Amiens University Medical Center, Amiens, France; ^2^AGIR Research Unit, UR UPJV 4294, Jules Verne University of Picardie, Amiens, France; ^3^ISP1282 INRA University of Tours, Tours, France; ^4^Université Lille, CNRS, INSERM, CHU Lille, Institut Pasteur de Lille, U1019-UMR 8204-CIIL-Center for Infection and Immunity of Lille, Lille, France

**Keywords:** SARS-CoV-2, COVID-19, spike, nucleocapsid, neutralizing antibodies, vaccine, convalescent plasma therapy

## Abstract

A better understanding of the anti-SARS-CoV-2 immune response is necessary to finely evaluate commercial serological assays but also to predict protection against reinfection and to help the development of vaccines. For this reason, we monitored the anti-SARS-CoV-2 antibody response in infected patients. In order to assess the time of seroconversion, we used 151 samples from 30 COVID-19 inpatients and monitored the detection kinetics of anti-S1, anti-S2, anti-RBD and anti-N antibodies with in-house ELISAs. We observed that specific antibodies were detectable in all inpatients 2 weeks post-symptom onset and that the detection of the SARS-CoV-2 Nucleocapsid and RBD was more sensitive than the detection of the S1 or S2 subunits. Using retroviral particles pseudotyped with the spike of the SARS-CoV-2, we also monitored the presence of neutralizing antibodies in these samples as well as 25 samples from asymptomatic individuals that were shown SARS-CoV-2 seropositive using commercial serological tests. Neutralizing antibodies reached a plateau 2 weeks post-symptom onset and then declined in the majority of inpatients but they were undetectable in 56% of asymptomatic patients. Our results indicate that the SARS-CoV-2 does not induce a prolonged neutralizing antibody response. They also suggest that induction of neutralizing antibodies is not the only strategy to adopt for the development of a vaccine. Finally, they imply that anti-SARS-CoV-2 neutralizing antibodies should be titrated to optimize convalescent plasma therapy.

## Introduction

The Severe Acute Respiratory Syndrome Coronavirus 2 (SARS-CoV-2) has recently emerged and caused a human pandemic of coronavirus disease 2019 (COVID-19) (Wu et al., 2020b; Zhou et al., 2020; Zhu et al., 2020). Most infected patients showed mild symptoms, but around 10% had severe symptoms, such as dyspnea, high respiratory rate, and low blood oxygen saturation which can lead to death due to respiratory or multiple organ failure. There is currently no specific treatment and vaccine and thus patients are treated with supportive care.

Among the coronaviruses structural proteins, the Spike (S) and the Nucleocapsid (N) proteins are the main immunogens (Meyer et al., 2014). The S protein consists of two subunits, S1 which contains the Receptor Binding Domain (RBD) and S2. Commercial SARS-CoV-2 serological assays that detect antibodies specific to these viral proteins/domains have become available but they need to be finely evaluated. Some manufacturers have decided to target the S1 and/or S2 subunits whereas others chose the RBD or the N protein. Furthermore, neutralizing antibodies (NAbs) are considered key to recovery and protection against viral disease but the SARS-CoV-2 NAb response remains poorly documented and it is still unknown how long cured patients will be protected against new infection (Kirkcaldy et al., 2020; Ota, 2020).

In this study, we aimed at monitoring the anti-SARS-CoV-2 antibody response in infected patients. Our results will help to better understand the SARS-CoV-2 humoral immune response and will be useful to evaluate commercial serological assays.

## Materials and Methods

### Study Population and Specimen

Thirty patients diagnosed SARS-CoV-2 positive by RT-PCR on a nasopharyngeal swab sample, between 25 February and 23 March 2020 at the Amiens University Medical Center, were enrolled in the study. The general information was extracted from electronic medical records and the clinical characteristics of the 30 inpatients are described in Supplementary Table 1. Inpatients were considered as having mild disease when needing non-intensive care or severe disease when needing intensive care. Samples from patients diagnosed positive for other human coronaviruses [OC43 (*n* = 5), 229E (*n* = 4), NL63 (*n* = 2) or HKU1 (*n* = 1)] were also tested as negative controls (Supplementary Table 2). Finally, we also used samples from 25 asymptomatic individuals (Table 1) that were shown SARS-CoV-2 seropositive using commercial serological tests (LIAISON^®^ SARS-CoV-2 IgG from DiaSorin and/or ELISA SARS-CoV-2 (IgG) from EUROIMMUN). All plasmas were decomplemented at 56°C for 30 min. The study was approved by the institutional review board of the Amiens University Medical Center (number PI2020_843_0046, 21 April 2020).

**TABLE 1:** SARS-CoV-2 asymptomatic patients included in the study.

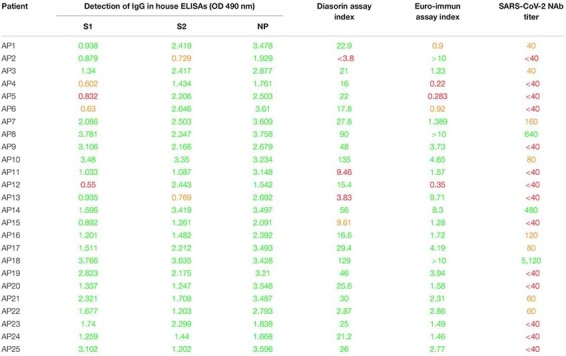

*Green, positive; Red, negative; Orange, equivocal. Red to green scale for low to high NAb titer (see Figure 5B).*

### In-House ELISAs

MaxiSorp Nunc-immuno 96-well plates were coated with a 1 μg/mL solution of SARS-CoV-2 S1, S2, RBD or N antigen (The Native Antigen Company, United Kingdom), overnight at 4°C. Wells were blocked with 1% fetal bovine serum for 1 h at 37°C. Then, 100 μL of diluted plasmas (1:100 for S1, S2 and RBD or 1:200 for N) were added and incubated for 1 h at 37°C. After washing 4 times, plates were incubated with peroxydase conjugated mouse anti-human IgG (Southern Biotech, 1/6,000). After 4 washes, 100 μL of o-phenylenediamine peroxidase substrate was added at room temperature in the dark. The reaction was stopped with H_2_SO_4_ solution 15 min later. The optical density was measured at 490 nm. All samples were run in triplicate. To establish the specificity of each assay, 40 pre-pandemic sera from 2019 were tested. Each cut-off values were defined as the means plus 3 standard deviations obtained with these samples.

### Neutralization Assay

Retroviral particles pseudotyped with the S glycoprotein of the SARS-CoV-2 (SARS-CoV-2pp) were produced as described previously (Millet and Whittaker, 2016), with a plasmid encoding a human codon-optimized sequence of the SARS-CoV-2 spike glycoprotein (accession number: MN908947). Supernatants containing the pseudotyped particles were harvested at 48, 72, and 96 h after transfection, pooled and filtered through 0.45 μm pore-sized membranes. Neutralization assays were performed by pre-incubating SARS-CoV-2pp and diluted plasma for 1 h at room temperature before contact with Vero cells (ATCC^®^ CCL-81^TM^) that were transiently transfected with the plasmids pcDNA3.1-hACE2 and pcDNA3.1-TMPRSS2 48 h before inoculation. Luciferase activities were measured 72 h post-infection, as indicated by the manufacturer (Promega). The NAb titers were defined as the highest dilution of plasma which resulted in a 90% decrease of the infectivity. Retroviral particles pseudotyped with the G glycoprotein of the Vesicular Stomatitis Virus (VSVpp) were used to control the specificity of the neutralization.

### Statistical Analysis

Quantitative variables were expressed as the median and compared using Student’s *t*-test. The Pearson correlation coefficient was used to measure the strength of a linear association between two quantitative variables. Statistical analyses were performed using GraphPad Prism 5. A two-sided *P*-value <0.05 was considered statistically significant.

## Results

### Antibody Response in SARS-CoV-2 Infected Inpatients

**FIGURE 1:**
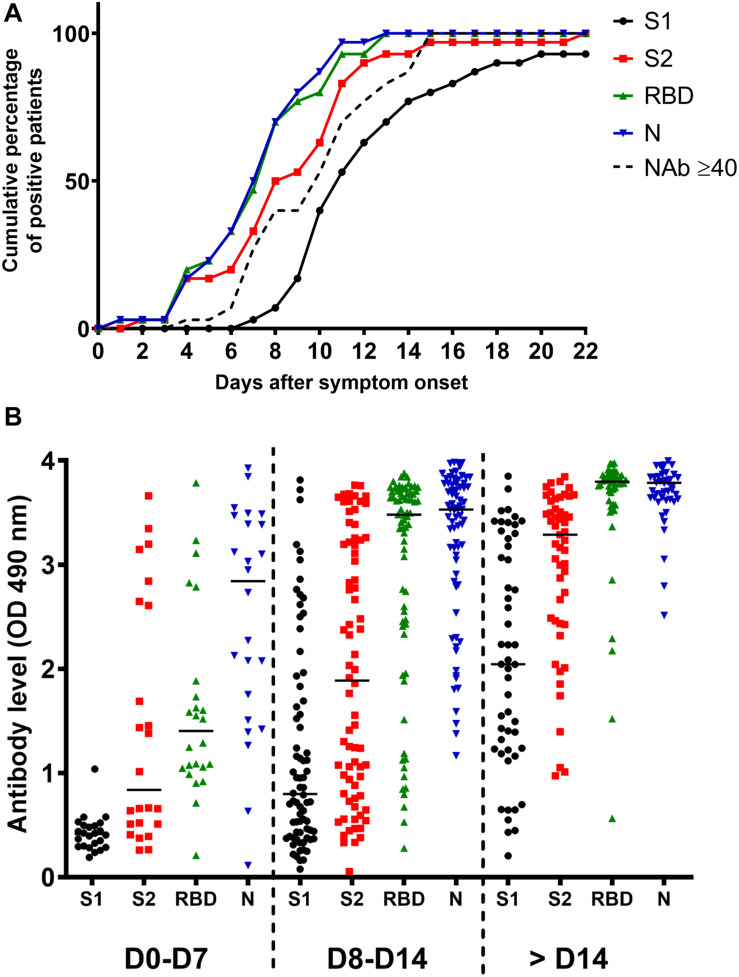
Antibody response in SARS-CoV-2 infected inpatients. **(A)** Kinetics of anti-S1, anti-S2, anti-RBD, anti-N and NAb detection in 30 COVID-19 inpatients post-symptom onset. **(B)** Evolution of the anti-S1, anti-S2, anti-RBD, and anti-N antibody levels during the first month post-symptom onset.

**FIGURE 2:**
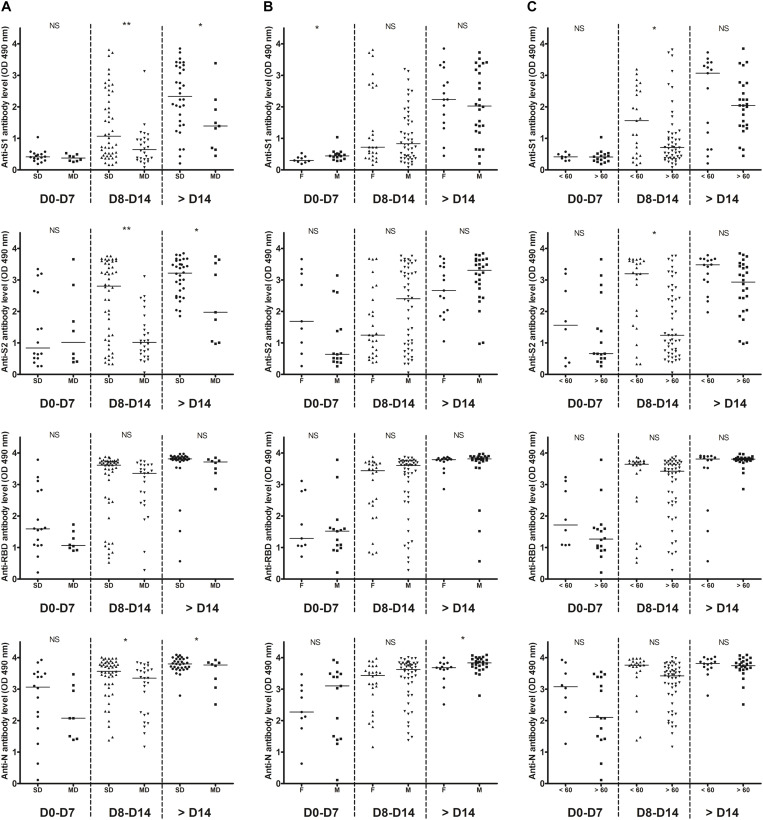
Temporal profiles of anti-S1, anti-S2, anti-RBD, and anti-N antibody levels. Inpatients samples were divided into three periods groups (day 0–7, day 8–14, and day > 14). **(A)** The temporal profiles are presented according to the severity of the disease (SD, severe disease requiring intensive care; MD, mild disease requiring non-intensive care). **(B)** The temporal profiles are presented according to the sex (M, male; F, female). **(C)** The temporal profiles are presented according to the age (< or >60 years old). Dashed lines indicate assays cut-offs for positivity and lines indicate the median for each assay. OD, optical density. NS, not significant; *p < 0.05; **p < 0.01.

In order to accurately assess the time of seroconversion, we used 151 samples from 30 patients hospitalized at the Amiens University Medical Center for a COVID-19 (see Supplementary Table 1) and monitored the kinetics of detection of anti-S1, anti-S2, anti-RBD and anti-N antibodies with in-house ELISAs. Importantly, plasmas from 12 patients that had previously been infected with other coronaviruses [OC43 (*n* = 5), 229E (*n* = 4), NL63 (*n* = 2) or HKU1 (*n* = 1)] showed minimal cross-reactivity, which highlights the specificity of these assays (Supplementary Table 2). We observed that antibodies targeting the N protein and the RBD were the earliest to be detected (Figure 1A). Thirteen days post-symptom onset, 100% of inpatients had detectable antibodies to both proteins. A similar profile was observed for anti-S2 antibodies but with a mean time lag of 2 days. Antibodies to the S1 subunit were the last to be detected and remained undetectable for two inpatients. High levels of anti-N and anti-RBD antibodies were detected in the large majority of samples obtained 14 days post-symptom onset whereas very heterogeneous levels of anti-S1 antibodies were found in the same samples (Figure 1B). The correlations between each ELISA are shown in Supplementary Figure 1 and clearly demonstrate that detection of the N protein and/or the RBD is more sensitive than the detection of the S1 (Supplementary Figures 1B,C) or the S2 subunit (see Supplementary Figures 1D,E). Anti-S1, anti-S2 and anti-N antibody levels were significantly higher in severe disease patients as compared to mild disease patients, from 8 days post-symptom onset (Figure 2A). A slight difference was observed for anti-N antibody levels according to the sex, from 14 days post-symptom onset (Figure 2B). Finally, a significant difference was observed for anti-S1 and anti-S2 antibodies according to the age, between 8 and 14 days post-symptom onset (Figure 2C).

### NAb Response to SARS-CoV-2 in COVID-19 Inpatients

**FIGURE 3:**
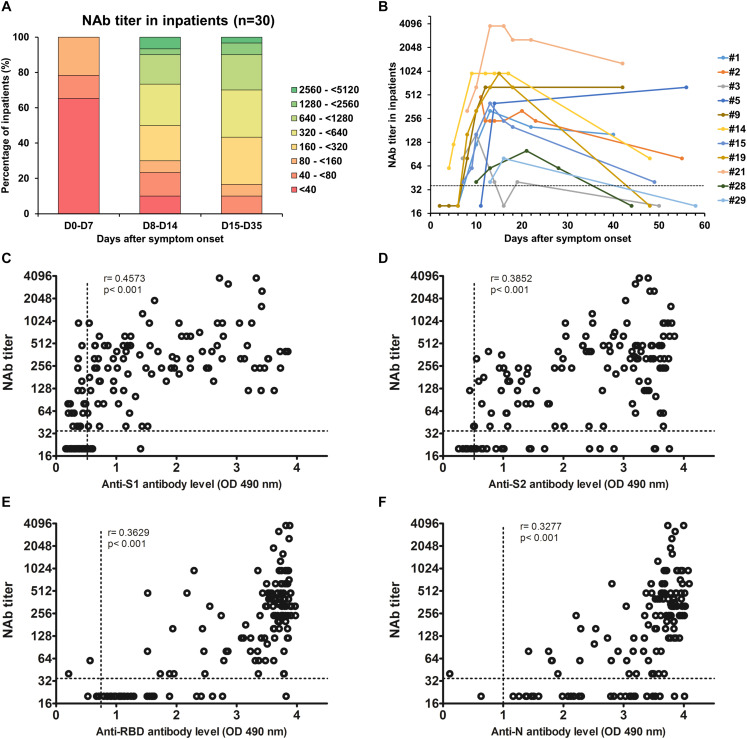
**(A)** Evolution of the NAb titer in 30 COVID-19 inpatients during the first month post-symptom onset. **(B)** Evolution of the NAb titer in 11 COVID-19 inpatients after more than 40 days post-symptom onset. The dashed line indicates the cut-off of the assay. **(C–F)** Correlations between NAb titers and anti-S1 **(C)**, anti-S2 **(D)**, anti-RBD **(E)**, and anti-N **(F)** antibody levels. Dashed lines indicate assay cut-offs for positivity. OD, optical density.

**FIGURE 4:**
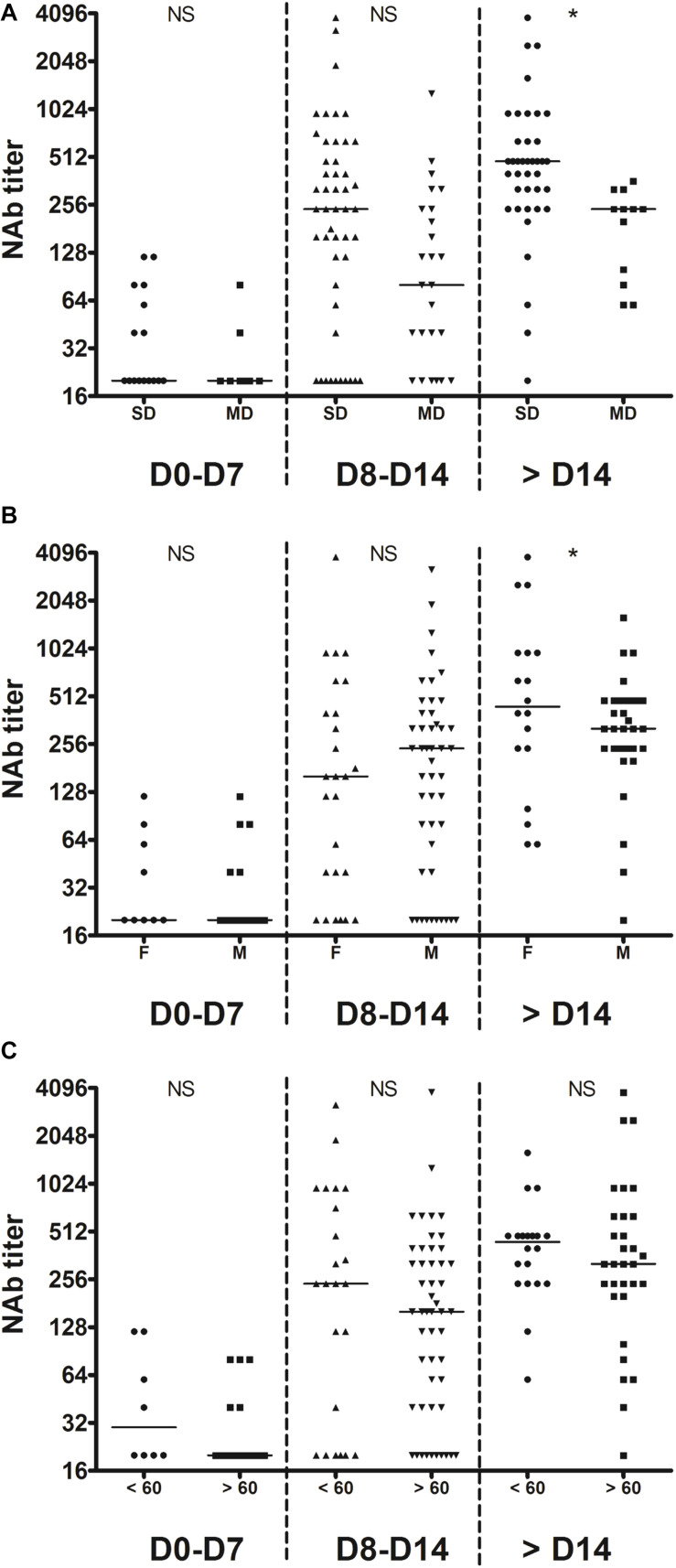
Temporal profiles of NAb titers. Inpatients samples were divided into three periods groups (day 0–7, day 8–14, and day >14). **(A)** The temporal profiles are presented according to the severity of the disease (SD, severe disease requiring intensive care; MD, mild disease requiring non-intensive care). **(B)** The temporal profiles are presented according to the sex (M, male; F, female). **(C)** The temporal profiles are presented according to the age (< or >60 years old). Dashed lines indicate assays cut-offs for positivity and lines indicate the median for each assay. NS, not significant; *p < 0.05.

We also monitored the presence of NAbs in all plasma samples using SARS-CoV-2pp (Millet and Whittaker, 2016). Importantly, several studies demonstrated that there was a significantly positive correlation in the NAb titers between such pseudotyped particles and the native SARS-CoV-2 (Liu et al., 2020; Ni et al., 2020; Schmidt et al., 2020). The results obtained for each inpatient are presented in Supplementary Figure 2. One sample of each inpatient was also used to perform dose-response curves with VSVpp and no inhibition was observed, demonstrating that the neutralization observed with the COVID-19 inpatient plasmas was specific to the SARS-CoV-2 (Supplementary Figure 3A). Furthermore, plasmas from the 12 patients that had previously been infected with other coronaviruses did not have any effect on SARS-CoV-2 pseudotype infectivity (Supplementary Table 2 and Supplementary Figure 3B). As expected, our results demonstrate that the NAb production kinetic correlates with the production of antibodies targeting the S1, S2 subunits as well as the RBD and we detected NAbs in all COVID-19 inpatients 15 days post-symptom onset (Figure 1A). The NAb titers increased from 1 week post-symptom onset and reached a plateau 1 week after (Figures 3A,B). However, the NAb titers reached were variable between inpatients, 17% generated low levels of NAbs (40 ≤ titers <160), 73% intermediate levels (160 ≤ titers <1,280) and 10% high levels (1,280 ≤ titers) (Figure 3A). We also had the opportunity to monitor the presence of NAbs in late samples of 11 inpatients (≥40 days post-symptom onset) and we observed that the NAb titer dropped to low or undetectable level in most of these samples (Figure 3B). We found poor correlations between NAb titers and anti-S1 (*r* = 0.4573), anti-S2 (*r* = 0.3852), anti-N (*r* = 0.3629) or anti-RBD (*r* = 0.3277) antibody levels (Figures 3C–F). Significantly higher NAb titers were observed in inpatients with severe forms (*p* = 0.04; Figure 4A) and in women (*p* = 0.03; Figure 4B) from 14 days post-symptom onset. In contrast, no significant difference was observed according to the age, probably because of the high heterogeneity of NAb levels in >60 years-old patients (Figure 4C). In addition, poor correlations were observed between NAb titers and white blood cells (*r* = 0.2384; Supplementary Figure 4A) as well as lymphocytes counts (*r* = 0.3696; Supplementary Figure 4B), suggesting that the amounts of NAb produced did not depend on the amount of immune cells.

### SARS-CoV-2 NAbs in Asymptomatic Patient Samples

**FIGURE 5:**
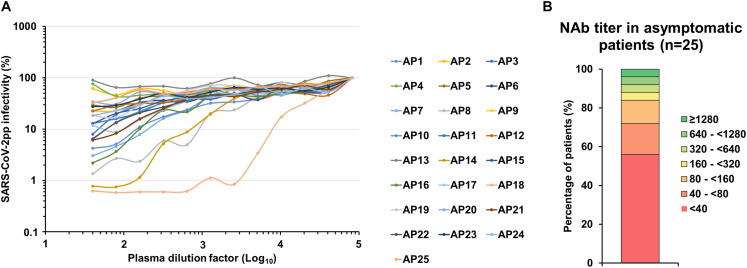
SARS-CoV-2 NAbs in asymptomatic individual samples. **(A)** SARS-CoV-2pp were pre-incubated with serially diluted plasma obtained from 25 asymptomatic patients that were seropositive with commercial serological assays (AP1 to AP25). Dose response curves represent the means of normalized infectivity (%) from two independent experiments performed in duplicate. Error bars have been omitted for clarity. **(B)** Determination of the NAb titer in plasma samples from 25 asymptomatic patients.

Finally, we had the opportunity to monitor the presence of NAbs in plasma samples from 25 individuals who had been asymptomatically infected with SARS-CoV-2 based on their positive results with commercial serological assays as well as in-house ELISAs (Table 1). It is important to note that we could not establish when these patients had been infected since they were asymptomatic but it probably occurred more than 1 week before sampling since they were seropositive and thus they had already produced antibodies. The results obtained for each patient are presented in Figure 5A and a synthesis is shown in Table 1 and Figure 5B. NAbs were below the detection limit of our assay (<40) in the majority of these plasma samples (56%, 14/25). Low NAb levels (40 ≤ titers <160) were found in 28% of these patients (7/25). Three patients had intermediate NAb levels (160 ≤ titers <1,280) and only one showed a high NAb titer (≥1,280).

## Discussion

Commercial serological assays that are complementary to direct viral detection of the SARS-CoV-2 by RT-PCR have recently become available but they need to be finely evaluated (Demey et al., 2020; Krüttgen et al., 2020). We only tested IgG detection since recent data showed that anti-SARS-CoV-2 IgG levels increase at the same time or earlier than IgM levels (To et al., 2020). With our four in-house ELISAs, we showed that the detection of the RBD and the N protein may be more suitable since it was highly or slightly more sensitive than the detection of S1 or S2, respectively. The presence of cryptic epitopes in the RBD could explain why in some cases anti-RBD antibodies are detected whereas anti-S1 antibodies are not (Yuan et al., 2020).

As other groups, we report that COVID-19 patients generate variable levels of NAbs that reach a plateau 2 weeks post-symptom onset (Fafi-Kremer et al., 2020; Liu et al., 2020; Ni et al., 2020; Robbiani et al., 2020; Wang X. et al., 2020; Wölfel et al., 2020; Wu et al., 2020a). Our results also indicate that the SARS-CoV-2 does not induce a prolonged NAb response since we observed a drop of the NAb titer for several patients a few weeks after infection. This is in agreement with observations by other groups (Seow et al., 2020; Wang K. et al., 2020; Wu et al., 2020a). For instance, Wu et al. (2020a) reported that the median NAb titer in plasma at follow-up 2 weeks post-discharge was significantly lower than that at the time of discharge, in a cohort of 117 patients who have recovered from mild COVID-19. We also report that patients requiring intensive care had an augmented NAb response compared to non-intensive care patients. In agreement with this result, other studies demonstrated that the magnitude of the NAb response is dependent upon the disease severity (Liu et al., 2020; Seow et al., 2020). Furthermore, we observed that NAbs were undetectable in around half of asymptomatic patients. suggesting either that they had not been produced or that they had already declined. Accordingly, Ko et al. (2020) reported that most asymptomatic and mild COVID-19 patients produced NAbs, although the titers were lower than severe disease patients.

Altogether, our results raise questions concerning the role played by NAbs in COVID-19 cure and the longevity of the protection against reinfection. Nonetheless, Robbiani et al. (2020) suggested that, even though all individuals who recovered from COVID-19 do not have high levels of NAb, they all have rare but recurring RBD-specific antibodies with potent antiviral activity. Furthermore, we must keep in mind that immunity is not just antibodies and that other arms of the immune system may also play a major role in COVID-19 cure and protection against reinfection. In particular, the immunological memory will certainly protect against severe disease if reinfection would occur (Cox and Brokstad, 2020). Accordingly, a robust T cell immunity has recently been evidenced in convalescent individuals with asymptomatic or mild COVID-19 (Sekine et al., 2020). Thus, our results suggest that induction of NAb production might not be the only strategy to adopt for the development of a SARS-CoV-2 vaccine. Finally, since COVID-19 patients produce variable levels of short-lived NAb and since NAb titers poorly correlate with S1, S2, RBD or N binding, our results imply that anti-SARS-CoV-2 NAbs should be titrated to optimize convalescent plasma therapy (Chen et al., 2020; Roback and Guarner, 2020).

## Data Availability Statement

All datasets presented in this study are included in the article/Supplementary Material.

## Ethics Statement

The studies involving human participants were reviewed and approved by the institutional review board of the Amiens University Medical Center (No. PI2020_843_0046, 21 April 2020). Written informed consent for participation was not required for this study in accordance with the national legislation and the institutional requirements.

## Author Contributions

EB, GD, CF, SC, and FH designed the research and analyzed the data. J-LS collected clinical samples. BD extracted clinical information from electronic medical records. AT, SB, and JD provided key reagents. EB and FH performed the experiments and wrote the manuscript. All authors contributed to the article and approved the submitted version.

## Conflict of Interest

The authors declare that the research was conducted in the absence of any commercial or financial relationships that could be construed as a potential conflict of interest.
