# Supplementary material for: Anti-spike, Anti-nucleocapsid and Neutralizing Antibodies in SARS-CoV-2 Inpatients and Asymptomatic Individuals
Source: Front Microbiol. 2020 Oct 19;11:584251. doi: 10.3389/fmicb.2020.584251 (PMC7604306; doi:10.3389/fmicb.2020.584251)
Supplement: Supplementary file 1 [file Data_Sheet_1.docx]

Supplementary Material

# Supplementary Figures and Tables

## Supplementary Figures


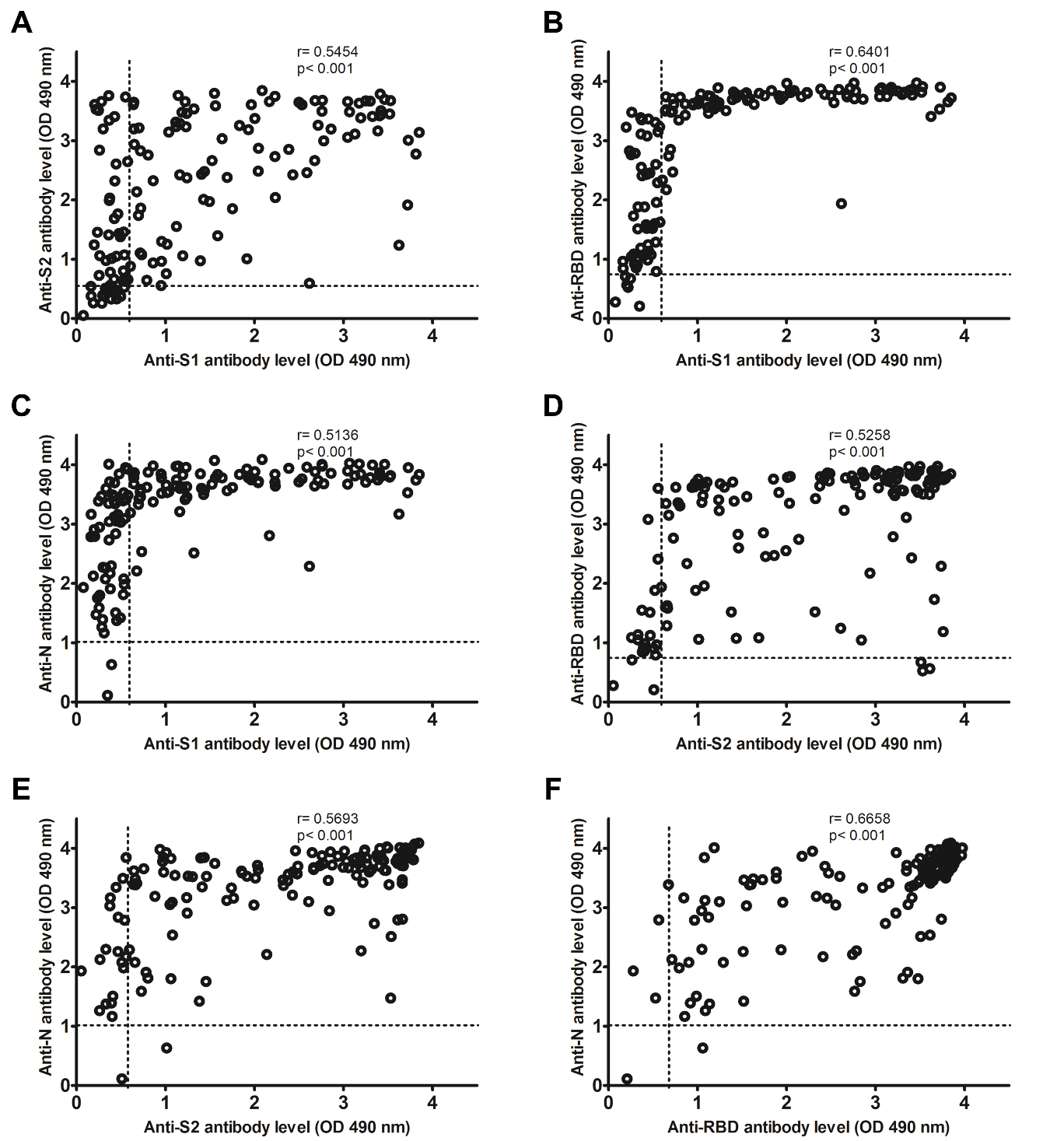


**Supplementary Figure 1 |** Correlations between anti-S1, anti-S2, anti-RBD and anti-N levels in in-house ELISAs. **(A)** anti-S1 versus anti-S2. **(B)** anti-S1 versus anti-RBD. **(C)** anti-S1 versus anti-N. **(D)** anti-S2 versus anti-RBD. **(E)** anti-S2 versus anti-N. **(F)** anti-RBD versus anti-N. Dashed lines indicate assay cut-offs for positivity. OD, optical density.


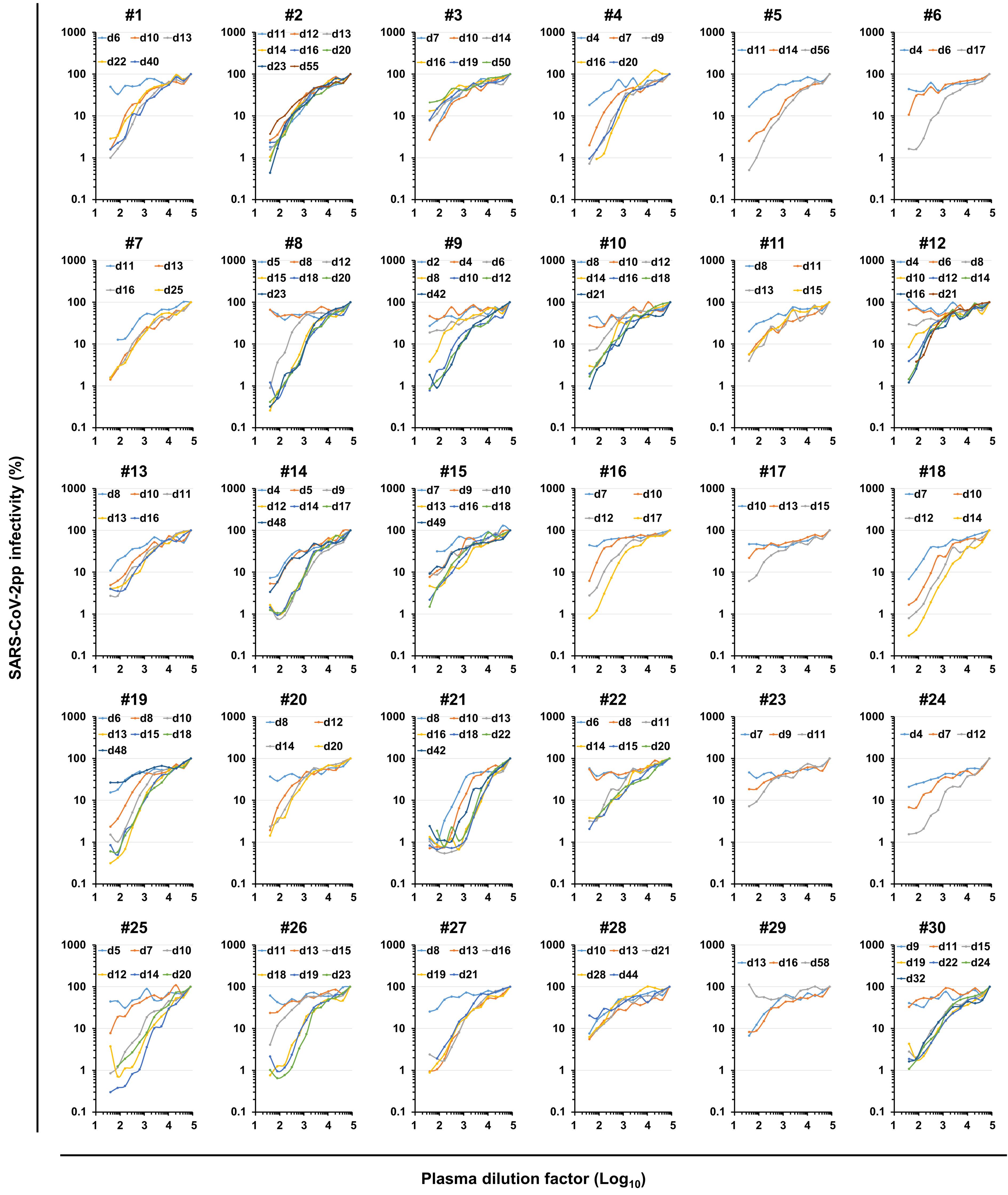


**Supplementary Figure 2 |** NAb response to SARS-CoV-2 in COVID-19 inpatients. SARS-CoV-2pp were preincubated with serially diluted plasma obtained from 30 COVID-19 inpatients (#1 to #30) at different days post-symptom onset (d2 to d58). Dose response curves represent the means of normalized infectivity (%) from two independent experiments performed in duplicate. Error bars have been omitted for clarity.


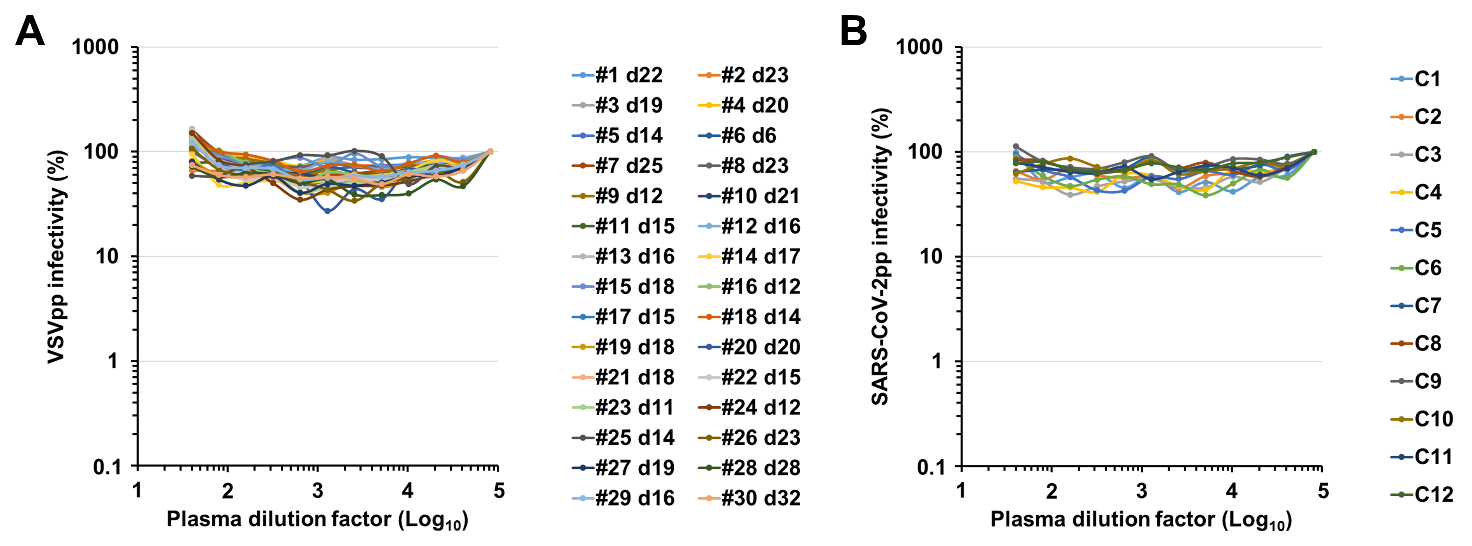


**Supplementary Figure 3.** Specificity of the neutralization assay. **(A)** VSVpp were preincubated with serially diluted plasma obtained from 30 COVID-19 inpatients (#1 to #30). **(B)** SARS-CoV-2pp were preincubated with serially diluted plasma obtained from 12 patients infected with 229E, NL63, HKU1 or OC43 coronaviruses (C1 to C12). Dose response curves represent the means of normalized infectivity (%) from two independent experiments performed in duplicate. Error bars have been omitted for clarity.


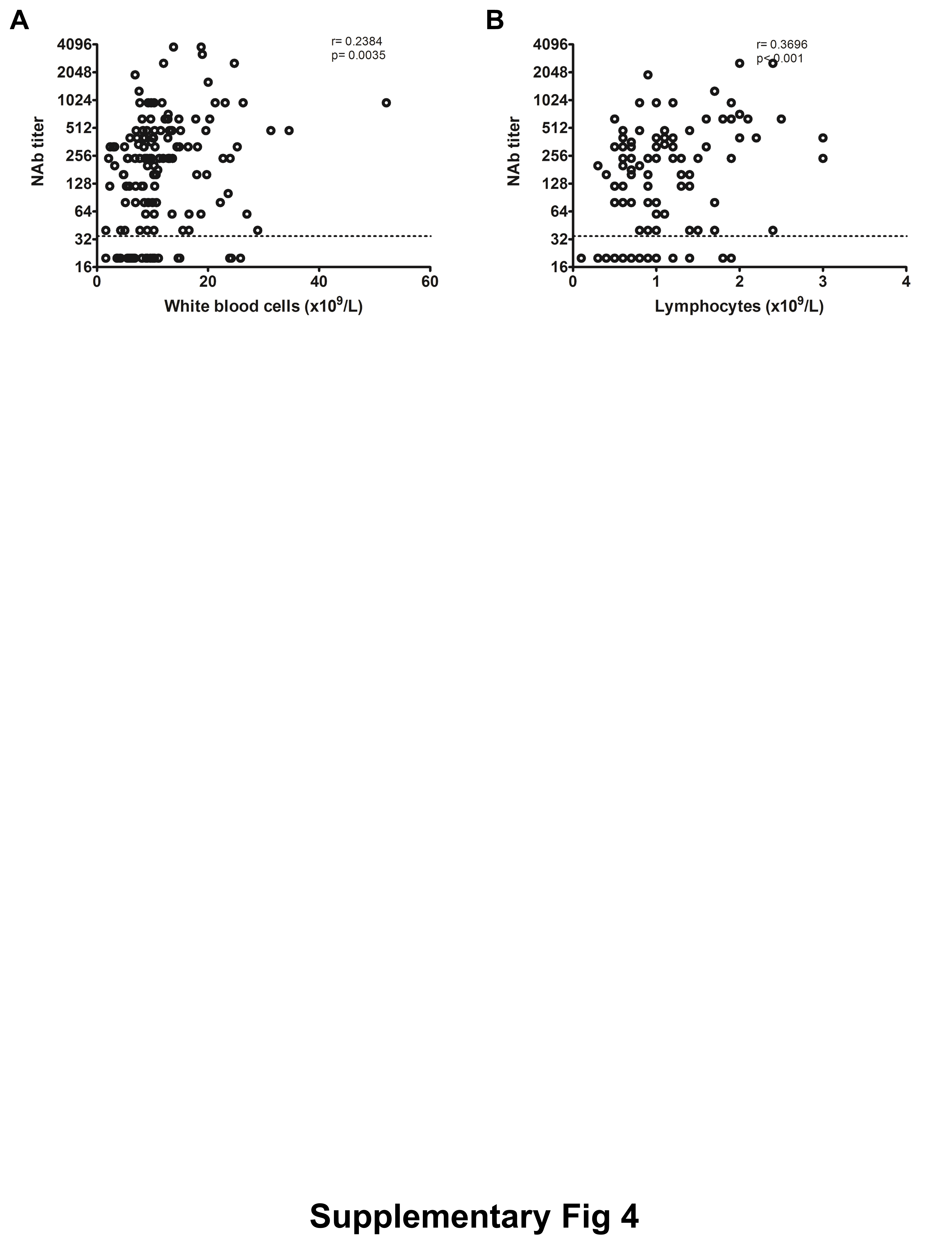


**Supplementary Figure 4 |** Correlations between NAb titers and white blood cells counts or lymphocyte counts. **(A)** NAb titer versus white blood cells counts. **(B)** NAb titer versus lymphocyte counts. Dashed lines indicate assay cut-offs for positivity. OD, optical density.

## Supplementary Tables

**Supplementary Table 1.** Characteristics of the 30 inpatients included in the study

| **Patient characteristics** | | **Mild Disease**  **(n=12)** | **Severe disease**  **(n=18)** |
| --- | --- | --- | --- |
| Female | | 6 (50%) | 6 (33%) |
| Male | | 6 (50%) | 12 (66%) |
| Median Age (Years) | | 77 (59-87) | 63 (38-78) |
| Chronic comorbidities | Hypertension | 7 | 9 |
|  | Chronic heart disease | 3 | 3 |
|  | Chronic lung disease | 3 | 0 |
|  | Chronic kidney disease | 2 | 0 |
|  | Diabetes | 3 | 4 |
|  | Hyperlipidemia | 2 | 1 |

**Supplementary Table 2.** Patients previously infected with endemic coronaviruses used as control in the study

| **Patient** | **Coronavirus strain** | **Date of the sample** | **Days post-diagnostic** | **Detection of IgG in our**  **in house ELISA assays** | | | | **SARS-CoV-2 NAb titer** |
| --- | --- | --- | --- | --- | --- | --- | --- | --- |
|  |  |  |  | **S1** | **S2** | **RBD** | **NP** |  |
| C1 | OC43 | 12/06/2019 | 174 | 0.398 | 0.424 | 0.763 | 0.425 | <40 |
| C2 | 229E | 26/04/2019 | 39 | 0.276 | 0.415 | 0.553 | 1.779 | <40 |
| C3 | NL63 | 11/09/2019 | 561 | 0.125 | 0.124 | 0.29 | 0.136 | <40 |
| C4 | OC43 | 27/06/2019 | 170 | 0.21 | 0.206 | 0.435 | 0.261 | <40 |
| C5 | OC43 | 28/06/2019 | 176 | 0.118 | 0.135 | 0.348 | 0.218 | <40 |
| C6 | NL63 | 04/03/2020 | 16 | 0.314 | 0.332 | 0.485 | 0.524 | <40 |
| C7 | 229E | 11/03/2019 | 14 | 0.303 | 0.323 | 1.034 | 1.865 | <40 |
| C8 | HKU1 | 16/03/2020 | 24 | 0.575 | 0.584 | 0.494 | 0.522 | <40 |
| C9 | OC43 | 01/08/2019 | 301 | 0.659 | 0.496 | 0.576 | 1.195 | <40 |
| C10 | 229E | 09/07/2019 | 56 | 0.354 | 0.324 | 0.524 | 0.74 | <40 |
| C11 | 229E | 03/04/2019 | 761 | 0.496 | 0.325 | 0.585 | 1.177 | <40 |
| C12 | OC43 | 28/02/2019 | 86 | 0.365 | 0.352 | 0.567 | 0.581 | <40 |

Green, positive ; Red, negative ; Orange, equivocal

Red to green scale for low to high NAb titer
